# Influence of DPH1 and DPH5 Protein Variants on the Synthesis of Diphthamide, the Target of ADP-Ribosylating Toxins

**DOI:** 10.3390/toxins9030078

**Published:** 2017-02-24

**Authors:** Klaus Mayer, Anna Schröder, Jerome Schnitger, Sebastian Stahl, Ulrich Brinkmann

**Affiliations:** Roche Pharma Research & Early Development, Large Molecule Research, Roche Innovation Center Munich, Nonnenwald 2, 82377 Penzberg, Germany; klaus.mayer.km1@roche.com (K.M.); Anna.Schroeder1@gmx.de (A.S.); jerome.schnitger@roche.com (J.S.); sebastian.stahl@roche.com (S.S.)

**Keywords:** pseudomonas exotoxin, diphtheria toxin, OVCA1, targeted therapy, immunotoxin, biomarker

## Abstract

The diphthamide on eukaryotic translation elongation factor 2 (eEF2) is the target of ADP-ribosylating toxins and -derivatives that serve as payloads in targeted tumor therapy. Diphthamide is generated by seven DPH proteins; cells deficient in these (DPHko) lack diphthamide and are toxin-resistant. We have established assays to address the functionality of DPH1 (OVCA1) and DPH5 variants listed in dbSNP and cosmic databases: plasmids encoding wildtype and mutant DPHs were transfected into DPHko cells. Supplementation of DPH1 and DPH5 restores diphthamide synthesis and toxin sensitivity in DPH1ko and DPH5ko cells, respectively. Consequently, the determination of the diphthamide status of cells expressing DPH variants differentiates active and compromised proteins. The DPH1 frameshift variant L96fs* (with N-terminal 96 amino acids, truncated thereafter) and two splice isoforms lacking 80 or 140 amino acids at their N-termini failed to restore DPH1ko deficiency. The DPH1 frameshift variant R312fs* retained some residual activity even though it lacks a large C-terminal portion. DPH1 missense variants R27W and S56F retained activity while S221P had reduced activity, indicated by a decreased capability to restore diphthamide synthesis. The DPH5 nonsense or frameshift variants E60*, W136fs* and R207* (containing intact N-termini with truncations after 60, 136 or 207 amino acids, respectively) were inactive: none compensated the deficiency of DPH5ko cells. In contrast, missense variants D57G, G87R, S123C and Q170H as well as the frequently occurring DPH5 isoform delA212 retained activity. Sensitivity to ADP-ribosylating toxins and tumor-targeted immunotoxins depends on diphthamide which, in turn, requires DPH functionality. Because of that, DPH variants (in particular those that are functionally compromised) may serve as a biomarker and correlate with the efficacy of immunotoxin-based therapies.

## 1. Introduction

Diphthamide is a conserved modified histidine on eukaryotic translation elongation factor 2 (eEF2) and the target of ADP-ribosylating Diphtheria toxin (DT) and Pseudomonas Exotoxin A (PE) [1,2,3,4,5]. Derivatives of these toxins are applied as cytotoxic payloads in targeted tumor therapy, their activity is strictly dependent on the presence of diphthamide [6,7,8,9,10]. The determination of the “diphthamide status” is therefore of relevance for the prediction of the susceptibility of tumor cells to targeted ADP-ribosylating toxins [11,12,13,14].

**Figure 1:**
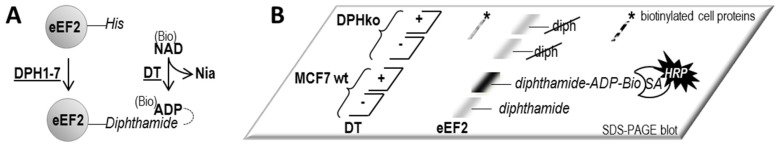
(**A**) Functionality of diphthamide synthesis genes determine His-eEF2 modification which, in turn, affects toxin-mediated ADP-ribosylation; (**B**) Exposure of cell extracts to DT and biotinylated NAD results in the bioADP-transfer and hence biotinylation of diphthamide-containing eEF2. The presence of diphthamide on eEF2 can thereby be detected by Western blot assays that detect biotinylated proteins with enzyme-conjugated streptavidin. Biotinylated cellular proteins are also detected and serve as the internal standard (loading control).

Synthesis pathways for the diphthamide modification and involved enzymes are as conserved among species as the diphthamide itself. Diphthamide is generated by the concert and successive action of seven proteins [1,15,16,17,18,19,20,21]: combined functionality of DPH1, DPH2, DPH3 and DPH4 is required to attach 3-amino-3-carboxypropyl (ACP) to H715 of human eEF2. This ACP-eEF2 becomes subsequently converted by the methyltransferase DPH5 to diphthine. The last synthesis step involves DPH6- and DPH7-dependent amidation of diphthine to diphthamide. Individual functionality of all these (non-redundant) diphthamide synthesis proteins (DPH1-7) is necessary to generate diphthamide and neither enzyme can compensate deficiency of another. Only diphthamide-eEF2 serves as target for ADP-ribosylating toxins (even the ‘almost complete’ diphthine modification is not recognized by toxins). Therefore, individual functionality of each DPH enzyme influences the diphthamide status of cells and thereby the potency of targeted toxins: reduced activity or loss of DPH functionality renders tumor cells less susceptible or resistant to ADP-ribosylating toxins and tumor-targeted immunotoxins ([1,11,14,15,21,22], Figure 1a).

Despite their high conservation and ubiquitous expression, limited information is available about the impact of variations or mutations on the functionality of DPH genes. Examples of DPH gene alterations include gene copy number changes (loss of heterozygosity, LOH) of the DPH1 (OVCA1) gene in ovarian cancers [23,24] or a deletion of the DPH7 (WDR85) gene. The latter (DPH7 deletion) caused toxin resistance in a lymphoma cell line [11]. Interference with the activity of diphthamide-dependent ADP-ribosylating toxins was also observed in cells whose DPH1 or DPH4 gene functionalities became reduced by the methylation of their respective promoters [14,15]. This indicates that DPH gene expression (modulated by gene copy number or promoter activity) affects the diphthamide synthesis and toxin sensitivity of tumor cells. In addition to expression-modulating variables, DPH genes harbor protein-altering variants, i.e., insertions, deletions, frameshift variants as well as amino acid exchanges. Many of these are listed in the Uniprot, dbSNP and Cosmic databases [25,26,27]. The impact of most of those variations on DPH protein functionality and their potential influence on immunotoxin therapy, however, has not been addressed. One reason for that appears to be the lack of reliable and robust assays to address the functionality of DPH variants, their impact on diphthamide generation, and on cellular sensitivity to targeted toxins.

Here, we describe a procedure for determining the functionality of DPH protein variants. DPH-deficient (DPHko) MCF7 cells lack diphthamide on eEF2 and are not ADP-ribosylated and hence are resistant to ADP-ribosylating toxins. Expression of the matching functional wildtype DPH protein in these cells re-establishes their diphthamide synthesis competency and restores toxin sensitivity. Functional and compromised DPH variants can be differentiated by evaluating their capability to compensate host cell deficiencies. The presence of diphthamide and toxin sensitivity can be determined via in vitro ADP-ribosylation assays (Figure 1b) that address the functionality of DPH1 or DPH5 variants in DPH1ko or DPH5ko cells, respectively. Functionally compromised DPH variants are candidates for response biomarkers for tumor therapies with PE- or DT-containing immunotoxins.

## 2. Results

### 2.1. Transient Expression of DPH1 and DPH5 cDNA in DPH Deficient MCF7 Cells

**Figure 2:**
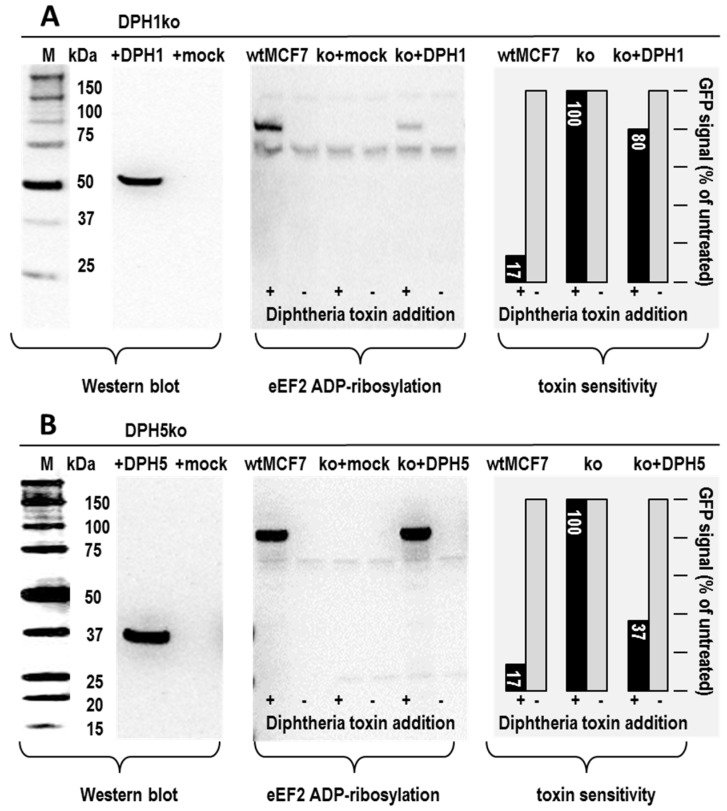
Expression of recombinant (**A**) DPH1 protein or (**B**) DPH5 protein in DPH-deficient MCF7 cells. DPH1 and DPH5 proteins are visualized by Western blot. DPH1-deficient cells are negative for DPH1 protein with full functionality of the other DPHs; DPH5-deficient cells are negative for DPH5 protein with full functionality of the other DPHs. DT-mediated ADP-ribosylation was detected by enzyme labeled streptavidin, as described in Figure 1. Cellular toxin sensitivity or resistance was assessed by GFP fluorescence in co-transfection assays (details in Materials and Methods).

Functionality of DPH1 and DPH1 variants can be assessed by recombinant expression in cells that by themselves lack DPH1 activity. In the same manner, the functionality and variants of DPH5 can be assessed in cells that lack DPH5. Plasmids harboring DPH1 or DPH5 cDNA expression cassettes were transfected into MCF7 cells which have both alleles of DPH1 or DPH5 inactivated and consequently lack DPH1 or DPH5 activity (DPHko cells, 22). These gene disruptions are close to the N-termini of the coding sequences, thus, DPHko cells do not express DPH protein. Therefore, DPH1 or DPH5 protein associated signals are absent in Western blots of cell extracts when probed with DPH1 or DPH5-protein specific antibodies. In contrast, cultures of DPH1ko or DPH5ko cells that are transfected with DPH1 or DPH5 expression plasmids contain DPH1 or DPH5 protein of expected size (left panels in Figure 2) while transfections with mock plasmids are DPH1 or DPH5 protein negative. Thus, transfection of DPH expression plasmids re-supplies DPH1 or DPH5 protein. Because the knockout cells are DPH1 or DPH5 deficient, all DPH1 or DPH5 protein in transfected cells is plasmid-encoded. Cells which receive plasmids that code for protein variants contain only the mutated DPH1 or DPH5 protein without activity of chromosomally encoded wild type protein.

### 2.2. Plasmid Encoded DPH Proteins Restore Diphthamide Synthesis and Toxin Sensitivity in DPH1 or DPH5 Deficient Cells

DPH1 and DPH5 are essential to generate diphthamide on eEF2, the sole target of ADP-ribosylating toxins. DPH1 and DPH5-deficient MCF7 are therefore resistant to ADP-ribosylation and cytotoxicity inflicted by toxins that target eEF2-diphthamide (22). Both parameters can be assessed to analyze the functionality of plasmid-encoded DPH proteins: in vitro ADP-ribosylation assays of eEF2-containing cell extracts detect the presence of diphthamide on eEF2; viability assays reveal the lethal consequences of toxin-mediated ADP-ribosylation of the diphthamide.

The results of in vitro ADP-ribosylation assays of MCF7 extracts are shown in the middle panels of Figure 2a,b: diphtheria toxin which targets diphthamide-eEF2 was applied to modify eEF2, using biotinylated NAD as substrate and enzyme-labeled streptavidin to detect ADP-ribosylated eEF2 (details in Materials and Methods and Figure 1). Wildtype MCF7 cells contain the diphthamide modification and therefore display toxin-mediated ADP-ribosylation of eEF2. In contrast, eEF2 of DPHko cells is not ADP-ribosylated as its eEF2 does not contain diphthamide. In a similar manner, toxin-treated extracts of DPHko cells that were transfected with control plasmids show no ADP-ribosylated eEF2.

On the other hand, toxin-mediated ADP-ribosylation of eEF2 is unambiguously detectable in extracts of DPH1ko cells (with defective chromosomal DPH1 genes) that were transfected with the DPH1 expression plasmid. In the same manner, toxin-mediated ADP-ribosylation of eEF2 was observed in extracts of DPH5ko cells (chromosomal DPH5 deficient) transfected with the DPH5 expression plasmid. Restauration of ADP-ribosylation as a consequence of DPH protein expression in DPHko cells was more pronounced for the DPH5ko/DPH5 combination than for the DPH1ko/DPH1 combination. These differences may be due to different recombinant expression or enzyme levels or enzyme kinetics. These in vitro ADP-ribosylation assays demonstrate that the plasmid encoded recombinant DPH1 and DPH5 proteins are functional and restore the diphthamide synthesis competency in their respective knockout cells.

Viability assays also differentiate between homogeneous cultures of wildtype and DPH ko cells, as cells which contain diphthamide are DT sensitive while diphthamide-deficient cells are resistant [22]. These assays, however, cannot be applied in the context of transfected cell populations because such cultures are a mixture of transfected and non-transfected cells. To overcome this limitation, DPH1- or DPH5-plasmids and GFP reporter-plasmids were co-transfected followed by toxin exposure and subsequent determination of GFP-fluorescence. Since the majority of transfected cells receive both plasmids, the co-transfection assays determine the viability of cells which have received the reporter plasmid as well as the DPH expression plasmid. Non-transfected cells do not express GFP and hence do not generate assay signals. Loss of viability of transfected cells is reflected by reduced fluorescence because cells of low viability or dead cells express less or no GFP. Figure 2 (right panels) shows that in alignment with previous reports, diphthamide-synthesis competent MCF7 wildtype cells are toxin-sensitive and DPH1ko or DPH5ko (diphthamide deficient) cells are toxin-resistant. Toxin resistance was also observed for mock-transfected (diphthamide deficient) DPHko cells. In contrast, evidence for re-sensitization was observed for MCF7-DPH1ko and DPH5ko cells upon transfection with the DPH1 or DPH5 encoding expression plasmids, respectively. Toxin-sensitivity as a consequence of DPH protein expression did not reach the full sensitivity levels of wildtype cells and was more pronounced for the DPH5ko/DPH5 combination than for the DPH1ko/DPH1 combination. These differences may be due to the different recombinant expressions or enzyme levels or kinetics, as well as due to the remaining unmodified eEF2 which is not amenable to toxin modification. Nevertheless, plasmid encoded DPH protein restores not only eEF2 diphthamide synthesis but also, to some degree, the toxin sensitivity of DPH-deficient cells. Thus, this assay confirms the functionality of recombinant DPH protein within cells, but it is clearly less sensitive and less robust than the in vitro ADP-ribosylation assay. Because of that, we applied the (robust) in vitro ADP-ribosylation assays to assess the functionality of DPH1 and DPH5 variants as described below.

### 2.3. DPH1 and DPH5 Variants

Variabilities of human genes, normal genomic variants as well as potential disease-associated mutations are listed in various databases, including Uniprot, dbSNP and COSMIC [25,26,27]. These databases list isoforms that change the master protein sequences of DPH1 and DPH5 (the master or “wildtype” sequences are defined herein by Uniprot Q9BZG8-1 and Q9H2P9-1). Some isoforms are described that change the size of the protein either by insertions or deletion of sequence stretches. A frequently occurring DPH5 isoform is also defined by one amino acid (ala212) deletion. In addition, numerous mutations/variants are listed for DPH1 as well as DPH5, each occurring with low frequency. Some of those cause premature termination or missense events that lead to non-conservative amino acid exchanges. The DPH1 and DPH5 isoforms and protein-changing variants whose functionality we have analyzed are listed in Table 1.

**Figure 3:**
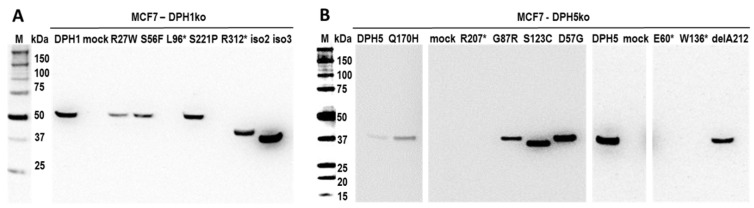
Expression of recombinant DPH proteins in DPH-deficient MCF7 cells. DPH proteins are visualized in Western blots in cells that express recombinant wildtype or variant DPH1 or DPH5 proteins. DPH-deficient cells are negative for the respective proteins. (**A**) expression of DPH1 and variants in DPH1-deficient cells; (**B**) expression of DPH5 and variants in DPH5-deficient cells. Some truncated DPH isoforms or frameshift variants are not detected due to size or instability (too small) or due to lack of recognition by the applied antibodies.

To address their functionality, DPH1 and DPH5 variants were recombinantly expressed in matching DPH-deficient cells. Recombinant DPH1 and DPH5 variants can be detected in extracts of cells transfected with amino acid variants (DPH1-R27W, -S56F, -S221P; DPH5-G87R, -S123C, -D57G, -Q170H, -delA212), as well as in cells that express the N-terminal truncated DPH1 isoforms 2 and 3 (Figure 3). In contrast, DPH1 or DPH5 associated signals could not be detected in mock-transfected cells (controls), or in cells that harbored plasmids encoding DPH1 or DPH5 variants with large truncations (DPH1-L96*, -R312*, DPH5-E60*, -W136*, -R207*). As the expression plasmids encoding those proteins was of correct (and otherwise identical) composition, the lack of detection is either due to failure to detect the antibodies applied in the Western blot, SDS-gel size limitations (E60*, L96*), or instability of the truncated abnormal proteins.

### 2.4. Activity of DPH1 and DPH5 Protein Variants

**Figure 4:**
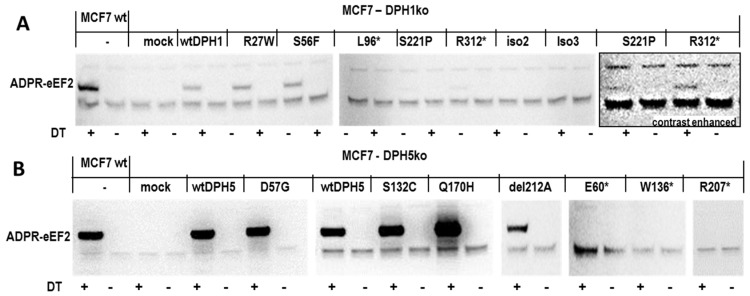
Capability of (**A**) DPH1 variants and (**B**) DPH5 variants to restore diphthamide synthesis deficiency of DPH1ko or DPH5ko cells, respectively. Extracts of cells expressing DPH variants were subjected to DT-mediated ADP-ribosylation followed by the detection of toxin-mediated modification of eEF2, as described in Figure 1. Extracts of MCF7 wildtype cells and of DPHko cells transfected with expression plasmids providing the functional wildtype enzyme (wtDPH1 and wtDPH5) serve as positive controls, extracts of mock-transfected cells and reactions without addition of DT serve as negative and specificity controls. Note that the right panel of (**A**) is a duplicate image of the S221P and R312* samples on the left, in a contrast enhanced setting to demonstrate the presence of weak but unambiguously detectable eEF2 bands. Additional ‘non-specific’ signals (unaffected by DT-modification) above and below eEF2 are biotin-containing cellular proteins.

To analyze the functionality of DPH1 and DPH5 protein variants, extracts of cells transfected with expression plasmids encoding the different proteins (see Figure 3) were subjected to in vitro DT-mediated ADP-ribosylation assays. Controls included extracts of wildtype MCF7 (diphthamide-eEF2) and of mock-transfected DPHko cells (eEF2 without diphthamide). All samples were processed in parallel under identical conditions. In addition, a parallel set of experiments was performed under identical conditions except for omitting DT to serve as the specificity control for toxin-dependence of ADP-ribosylation. The results of these assays for DPH1 and DPH1 variants are shown in Figure 4a: eEF2 of MCF7 wildtype cells and of DPH1ko cells transfected with unmodified DPH1 become ADP-ribosylated by the toxin. ADP-ribosylation does not occur without the toxin, confirming the specificity of the reaction. Extracts of mock-transfected DPH1ko cells also display no toxin-mediated ADP-ribosylation due to the lack of diphthamide on eEF2. By comparing the signals of DPH1 variants to these controls, we could assign qualitative ‘activity labels’ to the different DPH1 variants (our assay does not permit an exact quantification of activity): (i) “functional variants” are defined as those which modify eEF2 to such a level that it becomes ADP-ribosylated by the toxin to a similar degree as in wildtype-transfected extracts. Variants that fulfilled this parameter, i.e., they overcame the diphthamide synthesis deficiency of DPH1ko cells, included DPH1-R27W and DPH1-S56F; (ii) “reduced function variants” are defined as those which permit DT-mediated ADP-ribosylation, yet to a much lower level than observed in wildtype-transfected extracts. This phenotype was observed for DPH1-S221P and DPH1-R312* (note the contrast-enhanced insets in Figure 4A to display weak ADPR-signals; note also that we could not quantitate the expression level of R312* by our Western blot analyses); (iii) “loss of function variants” are defined as those which do not generate a detectable level of DT-mediated ADP-ribosylation. This phenotype was observed for the short truncated DPH1 variant L96* and the two truncated DPH1 isoforms 2 and 3.

Figure 4b shows the results of the analyses of DPH5 variants: eEF2 of MCF7 wildtype cells and eEF2 of DPH5ko cells transfected with unmodified DPH5 become ADP-ribosylated by the toxin. ADP-ribosylation does not occur without the toxin, confirming the specificity of the reaction. Extracts of mock-transfected DPH5ko cells also display no toxin-mediated ADP-ribosylation. Qualitative ‘activity labels’ were given to the different DPH5 variants as described above for DPH1. Functional variants whose eEF2 become modified to a similar degree as eEF2 of cells transfected with wildtype DPH5 were DPH5-D57G, DPH5-Q170H, DPH5-S123C and the DPH5-A212 deletion isoform. In contrast, all extracts of DPH5ko cells transfected with truncated variants of DPH5 (E60*, W136*, R207*) did not contain ADP-ribosylated eEF2. It has to be noted that neither of these ‘inactive’ isoforms could be detected as a recombinant protein in our cell extracts (see Figure 3). The inability to compensate DPH5 deficiency could therefore be attributable to the loss of enzymatic function (likely at least for E60* and W136*), an expression inability or rapid degradation, or to a combination of both.

## 3. Discussion

We have applied assays based on the recombinant complementation of DPH gene deficiency in tumor cells to address the functionality of DPH1 and DPH5 variants. The determination of diphthamide-ADP-ribosylation and cytotoxicity differentiated between functional and compromised protein isoforms or variants that are listed in gene variability databases (dbSNP, Cosmic, [25,26,27]).

DPH proteins are essential for generating eEF2-diphthamide which is the sole target of DT- and PE-derived immunotoxins. A lack of diphthamide renders tumor cells resistant towards tumor-targeted immunotoxins. Reduced levels of diphthamide may also render tumor cells less sensitive towards tumor-targeted immunotoxins. Functionality of DPH proteins does therefore affect cellular sensitivity and hence may serve as a response biomarker for immunotoxin therapy. So far, the relevance of DPH protein functionality as an efficacy predictor for targeted cancer therapy has been demonstrated in few examples which relate to reduced DPH gene expression. Promoter methylation of DPH1 and DPH4 genes correlated with reduced sensitivity of cells to targeted toxins that contain truncated derivatives of Pseudomonas Exotoxin A (PE) or DT as cytotoxic payload [13,14,15]. Also, recombinant tumor (and yeast) cells harboring DPH gene knockouts were resistant to PE, DT and immunotoxins, confirming the essentiality of DPH functionality for immunotoxin therapy [1,16,20,22]

Amino acid alterations and isoforms of DPH1 proteins are listed in gene variability databases. However, effects on protein functionality and the potential impact on the efficacy of targeted therapies has not been experimentally addressed for most variants. The human DPH1 gene is identical to OVCA1, for which copy number variations are known. Loss of heterozygosity is described in ovarian cancer for OVCA1 [20,23,24] which therefore has been proposed to be a tumor suppressor gene. The relevance of DPH1/OVCA1 gene copy number variation or of protein alterations on the efficacy of targeted therapy are unknown so far. Our analyses revealed that the amino acid exchanges inferred by most of the analyzed DPH1 missense mutations did not inactivate the DPH1 protein: The variations R27W and S56F were as capable as the wildtype protein in compensating DPH1 functionality and restoring toxin sensitivity. One of the missense variants (S221P) lost activity, as indicated by reduced toxin-mediated diphthamide modification in the extract. Mapping this variant onto a structure model of DPH1 indicates that this inactivation is probably caused by structural incompatibilities rather than due to mutation of the proposed active site of the enzyme.

The analyzed frameshift/nonsense variant L96* and the N terminal deleted isoforms 2 and 3 of DPH1 lost activity which is not unexpected as they lack large portions of the protein. One frameshift variant (R312*) surprisingly retained activity even though it lacks approximately 20% of the protein at its C-terminus. Although our assays are not suited to quantitatively assess if this protein is fully functional or if it has reduced activity, they nevertheless demonstrate that this variant has sufficient (remaining) activity to generate at least some diphthamide-eEF2 that becomes modified by toxins.

While DPH1 supports the first step in diphthamide synthesis (generation of eEF2-ACP), the DPH5 protein mediates the subsequent enzymatic step, i.e. conversion of ACP to diphthine. Except for analyses performed on recombinant cells lacking DPH5, the functionality of isoforms and protein variants and their impact on the efficacy of tumor-targeted ADP-ribosylating toxins is unknown. Our analyses differentiated between active and compromised DPH5 protein variants. All analyzed frameshift/nonsense variants and isoforms which lack large portions of the DPH5 protein (E60*, W136*, R207*) were unable to overcome DPH deficiency. In contrast, the missense variants D57G, G87R, S123C, Q170H as well as isoform delA212 of DPH5 (all of which correspond to gene deviations that are deposited in the dbSNP and Cosmic databases) retained activity.

Although the databases indicate the presence of a number of variants of DPH1 and DPH5 (and we identified some of them to be inactivating), their individual allele frequencies are quite low. Most of them (with the exception of some splice/frameshift/deletions and protein isoforms) were, in fact, observed as single occurrences. We have previously shown that only inactivation of all functional DPH1 or DPH5 alleles prevents diphthamide synthesis and mediates toxin resistance [22]. Retaining just one functional allele is sufficient to generate diphthamide and retain the toxin sensitivity of cells [22]. Because of that, rare variations in the DPH5 gene may not play a significant role in determining the sensitivity of tumor cells to targeted toxins (as inactivation of both functional alleles will be extremely rare). In contrast to rare DPH5 variations, infrequent DPH1 variations may affect the sensitivity of cells to (targeted) toxins. LOH of the DPH1/OVCA1 gene has been described in cancer, in particular in ovarian cancer [20,23,24]. Consequently, loss of one functional allele (and hence inability to compensate inactivating variations) makes it more likely that variations on the remaining allele modulate the cellular sensitivity to targeted toxins. Thus, variations or mutations that interfere with the functionality of the DPH1 may be biomarkers that associate with the response of cancer cells to targeted toxins that ADP-ribosylate the diphthamide on eEF2.

## 4. Materials and Methods

### 4.1. MCF7 Variants with Inactivated DPH Genes

DPH gene deficient MCF7 variants that have all the chromosomal copies of either the DPH1 gene or of the DPH5 gene inactivated were previously generated by S.Stahl et al. [22]. The cells lacking DPH1 or DPH5 enzyme activity are diphthamide-deficient and resistant to DT and PE, toxins which ADP-ribosylate the diphthamide on eEF2. The cells were grown in RPMI/10%FCS at 37 °C in humidified 5% CO_2_ conditions.

### 4.2. Expression of DPH Proteins

Recombinant expression of DPH proteins was achieved by transfecting MCF7 derivatives (deficient for the respective DPH-gene functionality) with plasmids containing expression cassettes for the CMV-promoter driven transient expression of DPH1 or DPH5. DPH protein was detected 24 h later in extracts of the transfected cultures by Western blot analyses. Antibodies that specifically detect the recombinant DDK-tagged DPH1 protein or the DPH5 protein were applied for the specific detection of the expressed DPH proteins.

### 4.3. ADP-Ribosylation of eEF2

Toxin-mediated ADP-ribosylation of diphthamide eEF2 was addressed in vitro in total cell extracts of transfected or non-transfected MCF7 or MCF7DPHko cells as previously described [22]. These cells were lysed in RIPA buffer containing protease inhibitors on ice; a 10 uL extract was subsequently exposed to 200 ng DT and 5 uM biotinylated NAD in a total volume of 30 uL for 1 h at 25 °C. EEF2 in these extracts which contains diphthamide becomes biotinylated via the transfer of biotinylated ADP; eEF2 without diphthamide is not modified/biotinylated by the toxin. The extracts are subsequently subjected to reducing SDS-PAGE, blotted onto membranes. Biotinylated eEF2 (indicative of the presence of diphthamide) is visualized as 100 kDa protein by probing with streptavidin-HRP and peroxidase substrate.

### 4.4. Cytotoxicity Assays

GFP-co-transfection based cytotoxicity assays were performed by co-transfecting DPH-expression plasmids and GFP expression plasmids into DPHko cells, followed by exposure to lethal toxin concentrations and the fluorescence-based detection of surviving GFP-expressing cells thereafter. Therefore, pCMV-GFP and pCMV-DPH1 or −5 expression plasmids and variant or mock (pCMV) plasmids were transfected into MCF7-DPHko cells using Lipofectamine according to the manufacturer’s protocol (Invitrogen, Thermo Fischer Sci., Waltham, MA, USA), 2500 ng plasmid/well in a 6-well format with equimolar ratio of GFP coding pCMV-GFP and pCMV-DPH test plasmids). After 24 h, the transfected cell pools were trypsinized and re-seeded at densities of 5000 cells/well in 96-well plates. The cells were allowed to settle for 6–8 h, subsequently Diphtheria Toxin was added to a final concentration of 50 nM, a dose that is lethal to MCF7 wildtype cells. Two to three days after toxin addition, the medium was removed, the wells were gently washed with 100 uL PBS to remove detached dead cells, and 100 uL PBS was added to each well. The GFP-mediated fluorescence of cells that remained on the wells after toxin exposure and wash was determined with a Microtiter reader (Infinite M200 PRO, Tecan) with 488 nm excitation and GFP-detection with a filter that detects emission at 522 nm. Per well, 12 overlapping ‘images’ were acquired and mean values were calculated from these. All assays were performed with at least triplicate wells/data set. Because dead or severely compromised cells produce no or very low GFP, GFP-signals can be taken as a measure to address the viability of co-transfected and hence also recombinant DPH expressing cells (irrespective of the presence of untransfected cells in the culture). Wells that received lethal (5 uM final conc.) doses of Staurosporine, instead of DT, defined background signals set to ‘0’. Wells that received a cell culture medium, instead of DT, defined 100% viability values.

## Figures and Tables

**Table 1:** Database-ID and composition of DPH1 and DPH5 isoforms and variants that we have analyzed. Expression levels of variants of DPH1 and DPH5 recombinantly produced in matching DPHko cells were estimated based on Western blot signals (Figure 3), and are listed in relation to cells that express recombinant wildtype protein. * Western blot detection does not permit exact quantification; ? some truncated/frameshift variants cannot be assessed because they are not detected in Western blots. Note that functionality correlates with detectable expression yet does not correlate with expression levels. DPH1-S221P is higher expressed than R27W or S56F yet with lower functionality, DPH5-Q170H is expressed to lower levels than D57G or S123C, yet displays the same functionality. Thus, functional differences are attributable to protein variants and not to differences in expression levels.

Protein	Variant	Type	Source	Expression *	Phenotype
DPH1	-	reference sequence (wt)	Uniprot Q9BZG8-1	+++	functional
	isotype 2	*N*-term deletion (1-80)	Uniprot Q9BZG8-2	++	loss of function
	isotype 3	*N*-term deletion (1-140)	Uniprot Q9BZG8-3	+++	loss of function
	R27W	missense	COSM 178273	+	functional
	S56F	missense	COSM 389195	+	functional
	S221P	missense	COSM 1381407*	++	reduced function
	L96 *	frameshift-stop	COSM 190903	?	loss of function
	R312 *	nonsense-stop	COSM 137079	?	reduced function
DPH5	-	reference sequence (wt)	Uniprot Q9H2P9-1	+-+++	functional
	del A212	single aa deletion isoform	Uniprot Q9H2P9-6	++	functional
	D57G	missense	COSM 893022	+++	functional
	G87R	missense	dbSNP rs376902046	++	functional
	S123C	missense	COSM 4792156	+++	functional
	Q170H	missense	COSM 674720	+	functional
	E60 *	nonsense-stop	COSM 260571	?	loss of function
	W136 *	frameshift-stop	COSM 5002373	?	loss of function
	R207 *	nonsense-stop	COSM 3417782	?	loss of function
